# A Role for the Hippo/YAP1 Pathway in the Regulation of In Vitro Vasculogenic Mimicry in Glioblastoma Cells

**DOI:** 10.1111/jcmm.70304

**Published:** 2024-12-24

**Authors:** Marie‐Eve Roy, Rahil Elimam, Alain Zgheib, Borhane Annabi

**Affiliations:** ^1^ Laboratoire d'Oncologie Moléculaire, Département de Chimie Université du Québec à Montréal Montreal Quebec Canada

**Keywords:** chemoresistance, glioblastoma, hippo pathway, vasculogenic mimicry, YAP/TEAD

## Abstract

The Hippo pathway plays a tumorigenic role in highly angiogenic glioblastoma (GBM), whereas little is known about clinically relevant Hippo pathway inhibitors' ability to target adaptive mechanisms involved in GBM chemoresistance. Their molecular impact was investigated here in vitro against an alternative process to tumour angiogenesis termed vasculogenic mimicry (VM) in GBM‐derived cell models. In silico analysis of the downstream Hippo signalling members *YAP1*, *TAZ* and *TEAD1* transcript levels in low‐grade glioblastoma (LGG) and GBM tumour tissues was performed using GEPIA. *TAZ* transcript levels did not differ between the healthy and tumour tissues data analysed. In contrast, *YAP1* transcript levels were elevated in GBM tissues, whereas *TEAD1* levels were high in both LGG and GBM. All three Hippo pathway inhibitors tested, GNE7883, VT107 and IAG933 effectively inhibited U87 and U251 cell migration and in vitro VM as assessed on Cultrex matrix. YAP1 gene and protein expression were induced upon VM, and its translocation to the nucleus was inhibited by the Hippo pathway inhibitors tested. SiRNA‐mediated transient silencing of *YAP1* repressed cell migration, VM formation and *CTGF* and *Cyr61* transcription. In conclusion, targeting of VM using Hippo pathway inhibitors could help circumvent GBM chemoresistance and effectively complement other brain cancer treatments.

**Abbreviations**
BSA: bovine serum albumin
FDA: Food and Drug Administration
GBM: glioblastoma
GEPIA: Gene Expression Profiling Interactive Analysis
GTEx: genotype‐tissue expression
LGG: low‐grade glioblastoma
NaF: sodium fluoride
PPIA: Peptidylprolyl Isomerase A
SDS: sodium dodecyl sulphate
SEM: standard error of the mean
TAZ: transcriptional coactivator with PDZ‐binding motif
TCGA: The Cancer Genome Atlas
TEAD: transcriptional enhanced associate domain
VM: vasculogenic mimicry
YAP1: yes‐associated protein 1

## 1 Introduction

The Hippo pathway is a key growth control pathway, in which downstream effectors, YAP (yes‐associated protein) and TAZ (transcriptional coactivator with PDZ‐binding motif), are frequently activated in cancers driving cell proliferation and tumour survival [1, 2, 3, 4]. Based on the premise that sustained interactions between YAP/TAZ and TEADs (transcriptional enhanced associate domain) are central to their transcriptional activities, recently discovered potent small‐molecule inhibitors that allosterically block the interactions between YAP/TAZ and all human TEAD paralogues through binding to their TEAD lipid pocket were developed and clinical trials initiated [5]. While Hippo pathway dysregulation is associated with various cancers [6, 7], impact of its specific targeting in highly invasive and angiogenic brain tumours such as glioblastomas (GBM) remains poorly understood. Pharmacological targeting of the chemoresistance phenotype associated with GBM further becomes highly relevant to also circumvent the molecular mechanisms associated with cancer treatment resistance [8, 9].

Recent studies have shown promising results in designing anticancer therapeutic strategies to target the Hippo pathway [10]. As such, preclinical studies suggest that these approaches can restore normal pathway function and suppress tumour development [11]. While the effectiveness of drugs targeting the Hippo pathway is currently being evaluated in clinical trials, overactivation of YAP/TAZ was found to contribute to uncontrolled cell growth and tumour progression in GBM [12, 13, 14], leading to therapy resistance through, in part, the maintenance of cancer stem cell population [15]. This further contributed to the immunosuppressive environment of GBM, making it difficult for the immune system to target and destroy cancer cells [16].

Vasculogenic mimicry (VM), a process believed to involve glioma stem‐like cells to generate vascular‐like structures that supply blood to sustain tumour growth and metastasis [17], is associated with poor clinical outcomes and resistance to anti‐angiogenesis therapies in various cancers, including GBM [18]. The molecular mechanisms underlying VM formation are complex and not fully understood, but recent evidence suggests that the Hippo pathway may play a key role in this process [19, 20]. In particular, YAP1 has been inferred to promote VM formation, migration, and invasion in hepatocellular carcinoma [19]. This evidence makes the Hippo pathway a potential target for anti‐tumour and anti‐metastasis therapies for inhibiting VM in GBM.

Several drugs have been granted an orphan drug designation by the U.S. Food and Drug Administration (FDA) and the European Medicines Agency (EMA) for the treatment of GBM [21, 22, 23]. Yet, there remains a high unmet need for new therapeutic strategies for GBM patients [24]. Recently, the development of LM98, a small‐molecule TEAD inhibitor derived from flufenamic acid was shown to reduce *Cyr61* and *CTGF* transcription, and led to the inhibition of triple‐negative breast cancer‐derived MDA‐MB‐231 cell migration and cell cycling arrest in the S phase [25]. Interestingly, *Cyr61* and *CTGF* basal transcript levels were found elevated in GBM tissues and were further upregulated when in vitro VM was monitored in U87 GBM cells [26]. Consequently, specific silencing of *Cyr61* and *CTGF* or treatment with LM98 impaired in vitro VM [26]. Concomitantly, development of LM41, AF2112 and HC258, the latter being a covalent acrylamide TEAD inhibitor, also strongly reduced the expression of *CTGF*, *Cyr61*, *Axl* and *NF2* [27, 28]. Given its role in tumour progression and chemoresistance, as well as metastasis in several cancers, therapeutic targeting of the Hippo pathway‐regulated VM may therefore hold promise against high‐grade GBM [12, 14].

In this study, we assessed the in vitro pharmacological properties of three clinically relevant TEAD binders that target the Hippo pathway to determine at the molecular level if they can circumvent VM‐mediated chemoresistance mechanisms in human GBM‐derived cell models. These inhibitors included GNE7883, a potent, reversible, allosteric inhibitor of the YAP1‐TEAD interaction [5], IAG933 that is currently in phase I clinical study in patients with mesothelioma, NF2/LATS1/LATS2‐mutated tumours and tumours with functional YAP1/TAZ fusions [11, 29] and VT107 currently being studied in clinical trials for its potential to inhibit TEAD auto‐palmitoylation in mesothelioma [30].

## 2 Materials and Methods

### 2.1 Materials

Sodium dodecyl sulphate (SDS) and bovine serum albumin (BSA) were purchased from Sigma‐Aldrich Corp (St Louis, MO, USA). Cell culture media EMEM was from Wisent (320‐005 CL). Electrophoresis reagents were purchased from Bio‐Rad Laboratories (Hercules, CA, USA). The HyGLO Chemiluminescent HRP (horseradish peroxidase) Antibody Detection Reagents were from Denville Scientific Inc. (Metuchen, NJ, USA). Micro bicinchoninic acid (BCA) protein assay reagents were from Pierce (Micro BCA Protein Assay Kit; Thermo Fisher Scientific, Waltham, MA, USA). The monoclonal antibodies against GAPDH (D4C6R, 97166) and YAP1 (1A12, 12395) were from Cell Signaling Technology (Danvers, MA, USA). The monoclonal antibody against Fibrillarin (NB300‐269) was purchased from Novus Biologicals (Toronto, ON). HRP‐conjugated donkey anti‐rabbit and anti‐mouse immunoglobulin (Ig) G secondary antibodies were from Jackson ImmunoResearch Laboratories (West Grove, PA, USA).

## 3 Methods

### 3.1 Cell Culture and Capillary‐Like Structure Formation Assay

The human U87 (HTB‐14), U118 (HTB‐15), U138 (HTB‐16), and U251 glioblastoma cell lines were from the American Type Culture Collection (ATCC, Manassas, VA, USA). They were all maintained in Eagle's Minimum Essential Medium (Wisent, 320‐006CL) containing 10% (v/v) calf serum (HyClone Laboratories, SH30541.03), 2 mM glutamine, 1 mM sodium pyruvate (Sigma‐Aldrich Canada, P2256), 100 units/mL penicillin and 100 mg/mL streptomycin (Wisent, 250‐202‐EL). Cells were incubated at 37°C with 5% CO_2_. VM was assessed in vitro using Cultrex (3432‐010‐01; R&D Systems Inc., Toronto, ON) to monitor 3D capillary‐like structure formation [31]. In brief, each well of a 96‐well plate was pre‐coated with 50 μL of Cultrex. Cell suspension in culture media (2 × 10^4^ cells/100 μL) was then seeded on top of polymerised Cultrex. Tested Hippo pathway inhibitors, IAG933, VT107 and GNE7883 were obtained from Chemietek (Indianapolis, IN, USA) and were added to the cell culture media at a 1 μM concentration and incubated at 37°C in a CO_2_ incubator. Pictures were taken over time using a digital camera coupled to a phase‐contrast inverted microscope. Mean loop area: For each loop, the area (number of pixels) enclosed by it is considered as its area. The mean loop area is the arithmetic mean of all loop areas. Mean loop perimeter: For each loop, the pixels that belong to its edge are considered its border or perimeter. The mean loop perimeter is the arithmetic mean of all loop perimeters. The number of loops and area covered upon tube branching formed by the cells were quantified using either the Wimasis analysis software (Cordoba, Spain) or the ImageJ software [32].

### 3.2 Total RNA Isolation, cDNA Synthesis and Real‐Time Quantitative PCR

Total RNA was extracted from cell monolayers using 1 mL of TriZol reagent for a maximum of 3 × 10^6^ cells as recommended by the manufacturer (Life Technologies, Gaithersburg, MD, USA). For cDNA synthesis, 1–2 μg of total RNA was reverse‐transcribed using a high‐capacity cDNA reverse transcription kit (Applied Biosystems, Foster City, CA, USA) or, in the case of the gene array, an R2 First Strand kit (QIAGEN, Valencia, CA, USA). The cDNA was stored at −80°C prior to PCR. Gene expression was quantified by real‐time quantitative PCR using iQ SYBR Green Supermix (Bio‐Rad, Hercules, CA, USA). DNA amplification was carried out using an Icycler iQ5 (Bio‐Rad) and product detection was performed by measuring the binding of the fluorescent dye SYBR Green I to double‐stranded DNA. The following primer sets were from QIAGEN: YAP1 (Hs_YAP1_1_SG, QT00080822), TEAD1 (Hs_TEAD1_1_SG, QT00000721), CTGF (Hs_CTGF_1_SG, QT00052899), CYR61 (Hs_CYR61_1_SG, QT00003451), GAPDH (Hs_GAPDH_1_SG, QT00079247) and Peptidylprolyl Isomerase A (PPIA; Hs_PPIA_4_SG, QT01866137). The relative quantities of target gene mRNA were normalised against internal housekeeping genes PPIA and GAPDH. The RNA was measured by following a ∆*C*_T_ method employing an amplification plot (fluorescence signal vs. cycle number). The difference (∆*C*_T_) between the mean values in the triplicate samples of the target gene and the housekeeping genes was calculated with the CFX manager Software version 2.1 and the relative quantified value (RQV) was expressed as 2^−∆*C*^_T_.

### 3.3 In Silico Analysis of TAZ, YAP1 and TEAD1 Transcripts Levels in Clinical Glioblastoma and Low‐Grade Glioma Tissues

A Gene Expression Profiling Interactive Analysis (GEPIA) web server was used to analyse the RNA sequencing expression data of glioblastoma tumours (GBM, *n* = 163) vs. healthy tissue (*n* = 207), and of low‐grade glioma (LGG, *n* = 518) vs. healthy tissue (*n* = 207) from The Cancer Genome Atlas (TCGA) and the normal brain tissue in Genotype‐Tissue Expression (GTEx) databases [33]. GEPIA provides customisable functions such as tumour/normal differential expression analysis, profiling according to cancer types or pathological stages, patient survival analysis, similar gene detection, correlation analysis and dimensionality reduction analysis (http://gepia.cancer‐pku.cn/detail.php, accessed on August 30th, 2024). One‐way ANOVA was used for differential analysis of gene expression using disease states (LGG, GBM) or healthy tissues as variables for the box plots.

### 3.4 Transfection Method and RNA Interference

For gene silencing experiments, U87 and U251 glioblastoma cells were transiently transfected with siRNA sequences using Lipofectamine‐2000 transfection reagent (Thermo Fisher Scientific, Waltham, MA, USA). Gene silencing was performed using 20 nM siRNA against *YAP1* (FlexiTube siRNA, SI02662954), or scrambled sequences (AllStar Negative Control siRNA, 1027281). The above small interfering RNA and mismatch siRNA were all synthesised by QIAGEN and annealed to form duplexes. Gene silencing efficacy was assessed by RT‐qPCR as described above.

### 3.5 Real‐Time Cell Migration Assay

Experiments were carried out using the Real‐Time Cell Analyser (RTCA) Dual‐Plate (DP) Instrument and the xCELLigence system (Roche Diagnostics, Laval, QC), following the instructions of the supplier. Cells were treated with vehicle (1% DMSO) or 10 μM of the tested Hippo pathway inhibitors. 2.5 × 10^4^ cells per well were seeded in a CIM‐plate 16 and incubated at 37°C under a humidified atmosphere containing 5% CO_2_ for 24 h. Prior to cell seeding, the underside of each well in the upper chamber was coated with 0.15% gelatine in PBS and incubated for 1 h at 37°C. The lower chamber wells were filled with either serum‐free medium or serum‐enriched medium. After 30 min of adhesion, cell migration was monitored every 5 min for 2 h. The impedance value was measured by the RTCA DP Instrument and expressed as an arbitrary unit called the cell index. Each experiment was performed in quadruplicate wells.

### 3.6 Nuclear Extraction

Cell monolayers were first lysed with a cytoplasmic buffer and then with a nuclear buffer according to the manufacturer's instructions (Invent Biotechnologies, SC‐003). In the case of cells cultured on Cultrex, they were first detached from the matrix using a non‐enzymatic Cultrex organoid harvesting and dissociation solution (3700‐100‐01; R&D Systems, Toronto, ON).

### 3.7 Western Blot

Cytosolic and nuclear fractions were isolated as described above. Total cell lysis was performed in a buffer containing 1 mM each of sodium fluoride (NaF) and sodium orthovanadate (Na_3_VO_4_). Proteins (10–20 μg) were then separated by SDS‐polyacrylamide gel electrophoresis (PAGE). Next, proteins were electro‐transferred to low‐fluorescence polyvinylidene difluoride membranes and blocked for 1 h at room temperature with 5% non‐fat dry milk in Tris‐buffered saline (150 mM NaCl, 20 mM Tris–HCl, pH 7.5) containing 0.3% Tween‐20 (TBST; Bioshop, TWN510‐500). Membranes were washed in TBST and incubated overnight with the appropriate primary antibodies (1/1000 dilution) in TBST containing 3% BSA and 0.1% sodium azide (Sigma‐Aldrich) at 4°C and on a shaker. After three washes with TBST, the membranes were incubated for 1 h with horseradish peroxidase‐conjugated anti‐rabbit or anti‐mouse IgG at 1/2500 dilutions in TBST containing 5% non‐fat dry milk. Immunoreactive material was visualised by ECL.

### 3.8 Statistical Data Analysis

Data and error bars were expressed as mean ± standard error of the mean (SEM) of three or more independent experiments unless otherwise stated. Hypothesis testing was conducted using the Kruskal–Wallis test followed by a Dunn Tukey's post‐test (data with more than three groups) or a Mann–Whitney test (two‐group comparisons). Probability values of less than 0.05 (*) were considered significant and denoted in the figures. All statistical analyses were performed using the GraphPad Prism 7 software (San Diego, CA, USA).

## 4 Results

### 4.1 Gene Expression Profiles of TAZ, YAP1 and TEAD1 in Four Human Glioblastoma Cell Lines and in Clinically Annotated Glioblastoma Tumour Tissues

**FIGURE 1:**
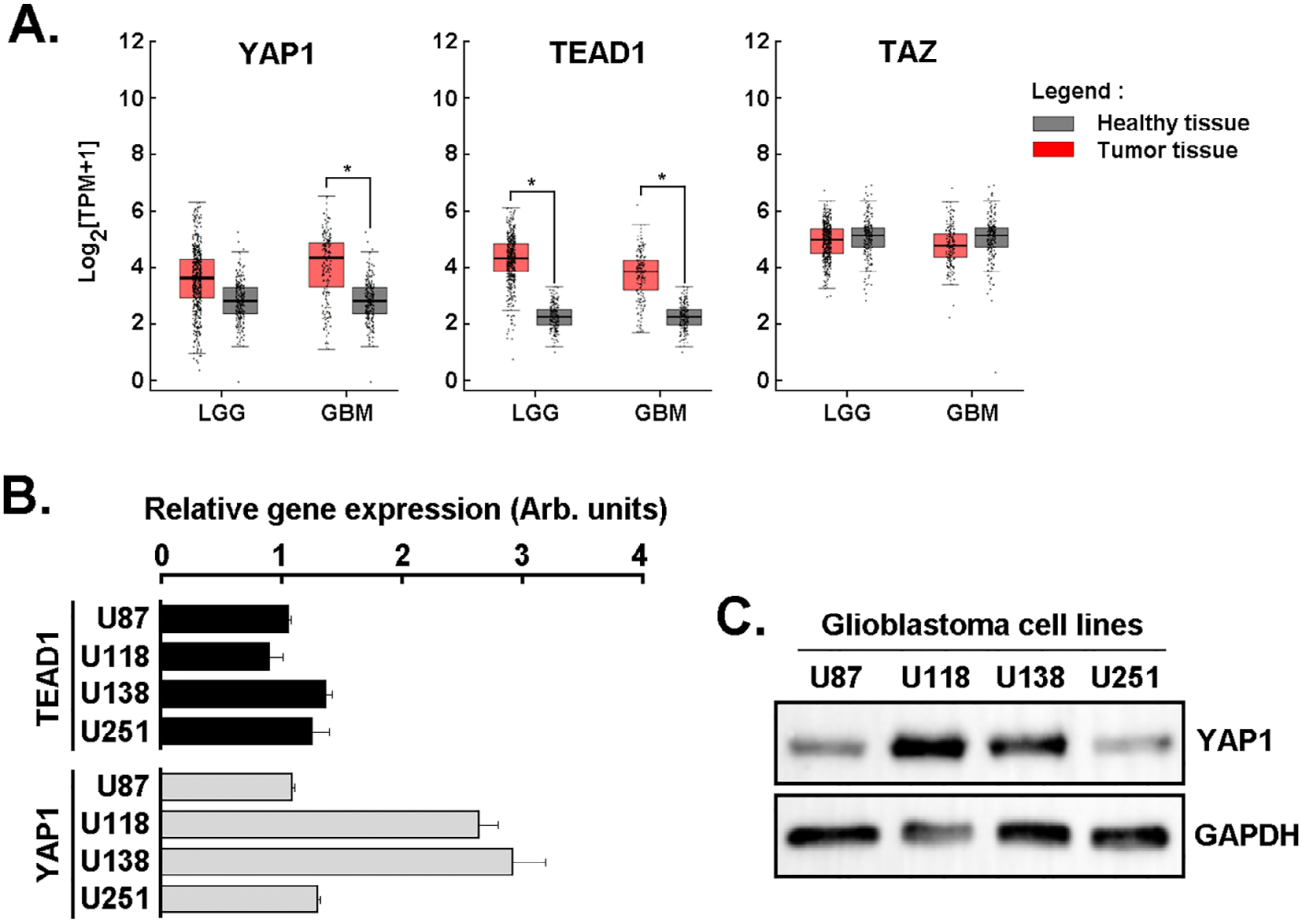
Gene expression profiles of TAZ, YAP1 and TEAD1 in four human glioblastoma cell lines and clinically annotated glioblastoma tumour tissues. (A) In silico analysis of *TAZ*, *TEAD1* and *YAP1* transcript levels was performed using RNA extracted from clinical samples from glioblastoma (GBM, *n* = 163) and low‐grade glioma (LGG, *n* = 518) (red boxes) and compared to healthy tissue (*n* = 207; grey boxes), (*p < 0.05). (B) Total RNA was extracted from four different human glioblastoma cell lines (U87, U118, U251 and U138) and relative gene expression profiles for *YAP1* and *TEAD1* determined by RT‐qPCR as described in the Methods section. (C) A representative immunoblot from two independent cell passages of YAP1 protein expression was performed from lysates of the four indicated GBM cell lines. GAPDH expression was assessed as a loading control.

TAZ, YAP1 and TEAD1 are key players in the Hippo signalling pathway in GBM where their expression and activation complex activates the transcription of genes involved in cell proliferation and survival [34]. YAP1 overexpression in GBM has additionally been linked to enhanced autophagy and lead to chemoresistance [35]. Here, transcript levels from 518 clinically annotated low‐grade glioma (LGG) and 163 GBM tissues were retrieved from the TCGA and from the normal brain tissue in GTEx databases and compared to 207 healthy tissues. *YAP1* and *TEAD1* transcript levels were effectively found to increase in GBM tissues, whereas those of *TAZ* remained unchanged among the healthy anti‐tumour tissue data analysed (Figure 1A). Increases in *YAP1* and *TEAD1* were reported previously to also correlate with increased levels of *Cyr61*, *CTGF* and *Axl* [26]. Interestingly, *YAP1* increases appeared specific to GBM whereas *TEAD1* was found increased in less invasive LGG as well, making it a less specific biomarker to distinguish between LGG and GBM. This observation therefore suggests a specific role for YAP1 in the more aggressive stage that GBM represents. *YAP1* and *TEAD1* gene expression profile was validated by a single amplicon amplification (not shown) and quantified by RT‐qPCR in four different human GBM‐derived cell line models, namely the U87, U118, U138 and U251 cells (Figure 1B). YAP1 was further found to be significantly expressed at the protein level in these four cell lines and coherent with gene expression (Figure 1C).

### 4.2 Hippo Pathway Clinical Inhibitors Alter the Chemotactic Cell Migration of Human U87 and U251 Glioblastoma Cells

**FIGURE 2:**
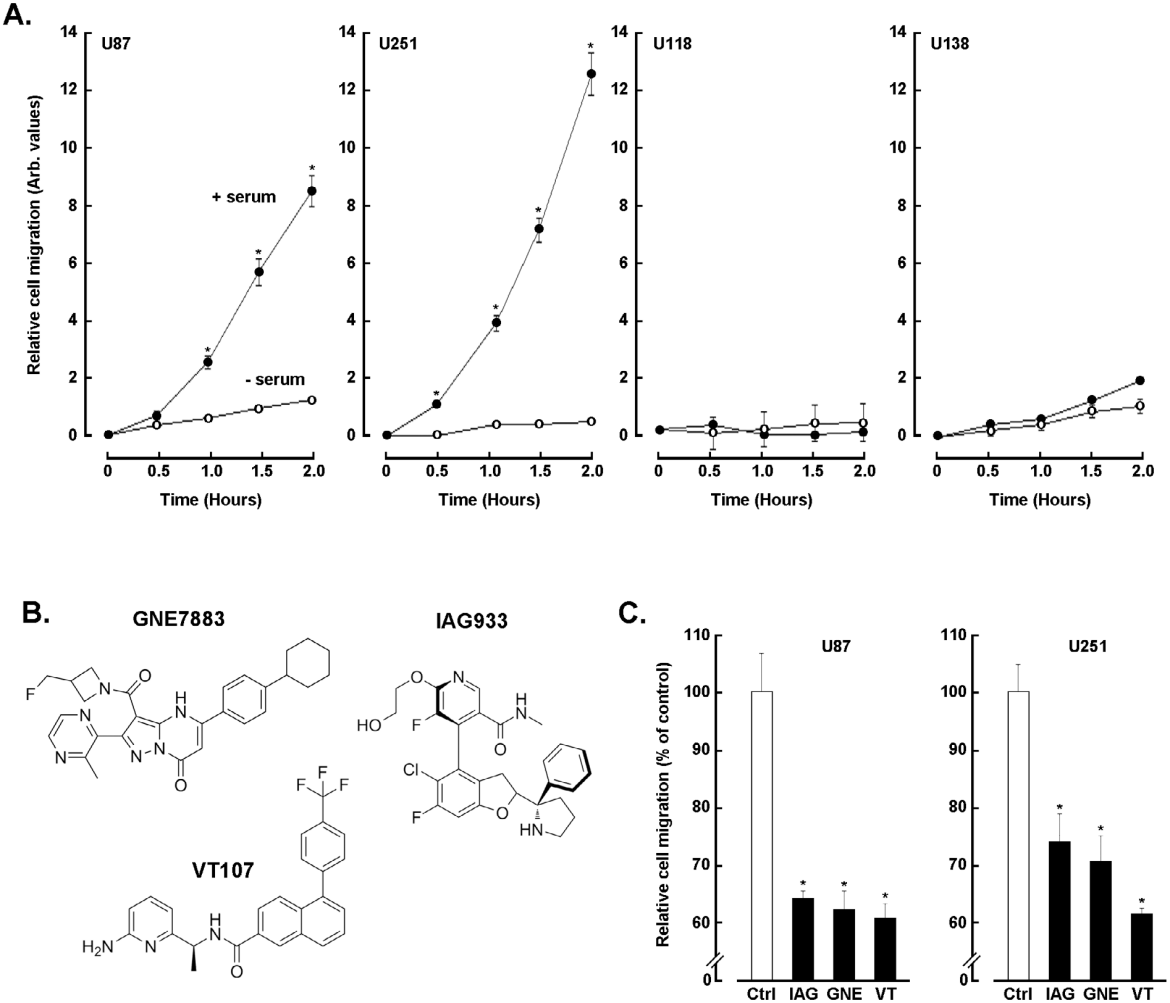
Hippo pathway clinical inhibitors alter the chemotactic cell migration of human U87 and U251 glioblastoma cells. (A) Real‐time cell chemotactic migration was monitored for 2 h for the four human glioblastoma cell lines indicated in the absence (open circle) or presence (closed circle) of serum. (B) Chemical structures of the three Hippo pathway clinical inhibitors tested, IAG933, GNE7883 and VT107. (C) Real‐time cell chemotactic migration in response to serum was monitored in U87 and U251 glioblastoma cell lines for 2 h in the absence (control, white bar) or presence of 10 μM of the Hippo pathway inhibitors indicated (black bars) (*p < 0.05).

Recent evidence has shown that targeting the YAP‐TEAD interaction can be a promising therapeutic strategy for GBM [36]. Here, clinically relevant Hippo pathway pharmacological inhibitors IAG933, GNE7883 and VT107 (Figure 2B) were screened against four different human GBM‐derived cell line models. Real‐time cell migration shows that serum‐mediated chemotaxis (Figure 2A, closed circle) was required to monitor significant migration in U87 and U251 cells, whereas in the absence of serum, very low chemotaxis was observed (Figure 2A, open circle). Intriguingly, virtually no response to serum was observed in U118 and U138 cells. When U87 and U251 cells were treated with any of the three Hippo pathway inhibitors, a significantly reduced relative cell migration was observed ranging from 25% to 40% inhibition (Figure 2C). Given their migration profile and response to Hippo pathway inhibition, these two cell line models were next further investigated for their involvement in in vitro VM.

### 4.3 Hippo Pathway Clinical Inhibitors Alter In Vitro Vasculogenic Mimicry of Human U87 Glioblastoma Cells

**FIGURE 3:**
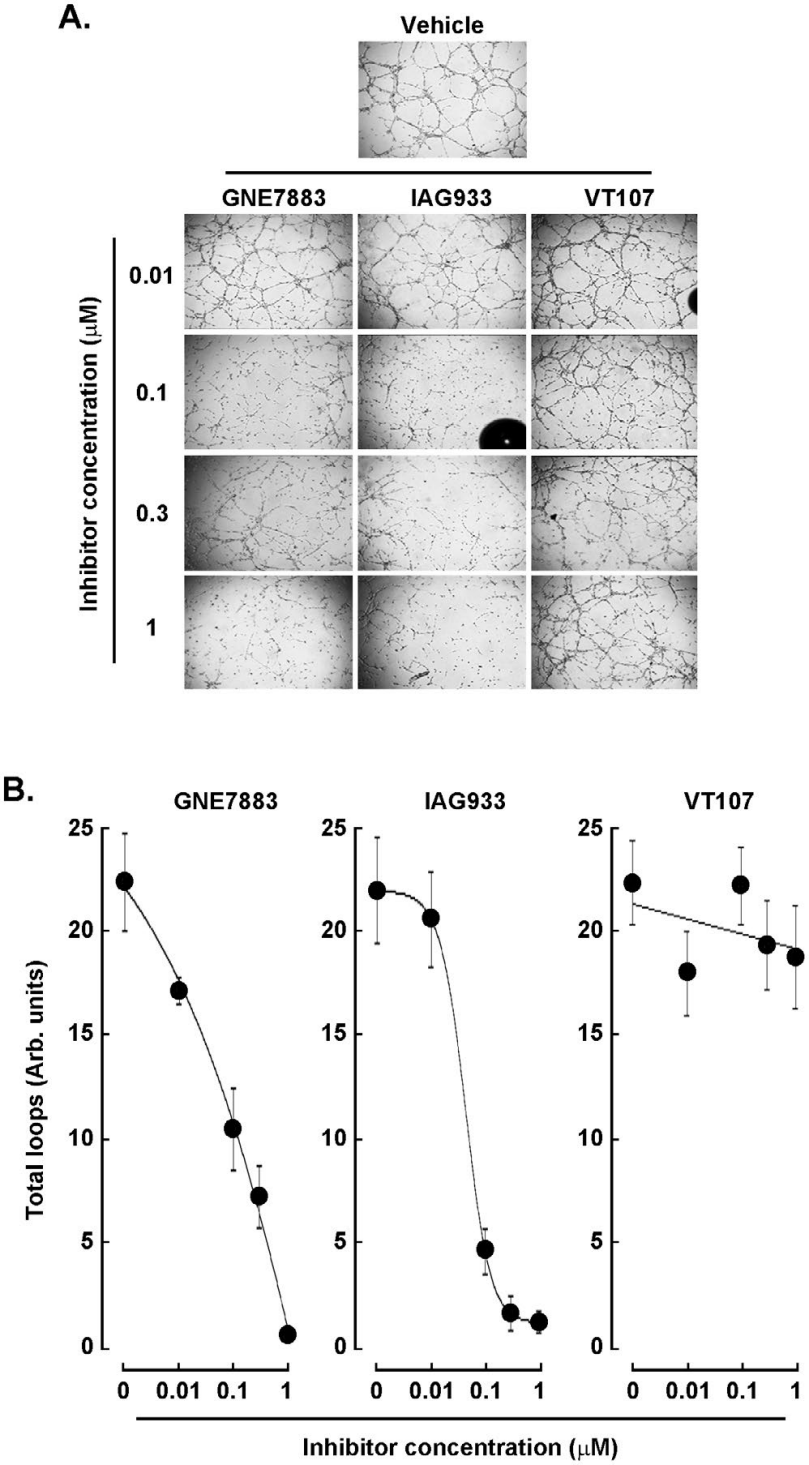
Hippo pathway clinical inhibitors alter in vitro vasculogenic mimicry of human U87 glioblastoma cells. U87 glioblastoma cells were trypsinised and seeded on top of Cultrex to generate 3D capillary‐like structures as described in Section 3. (A) Representative phase contrast pictures were taken to monitor structure formation at 24 h in the presence of the indicated concentrations of GNE7883, IAG933, and VT107, 4× magnification. (B) Total loop parameters were extracted from the Wimasis analysis of (A) and quantification was provided from a representative experiment performed in triplicate.

VM is particularly notable in GBM and is associated with poor prognosis [18, 37]. Such a process represents a significant challenge in GBM treatment as tumour cells form vessel‐like structures that facilitate blood supply independently of endothelial cells [38]. Clinical targeting of VM implies that one aims at circumventing the hypoxic tumour's ability to sustain itself and resist anti‐angiogenic therapies, which target traditional blood vessel formation. Given that targeted Hippo pathway treatments inhibited U87 and U251 cell migration, in vitro VM was next assessed. U87 cells were seeded on top of Cultrex and 3D capillary‐like structures formed as described in the Methods section (Figure 3A, upper panels). Increasing concentrations of GNE7883 and IAG933 were found to alter VM structures with IC_50_ values ranging from 0.04 to 0.1 μM in line with previously reported data (Figure 3B). VT107, within that time frame, did not alter VM structures and required over 48 h to exert its anti‐VM inhibitory effect (not shown). Along with their ability to inhibit chemotactic cell migration, the tested Hippo pathway inhibitors also altered VM structures in the U251 GBM‐derived cell line model (not shown).

### 4.4 In Vitro VM Triggers Nuclear YAP1 Expression in U87 and U251 Glioblastoma Cells and Is Inhibited by Hippo Pathway Pharmacological Targeting

**FIGURE 4:**
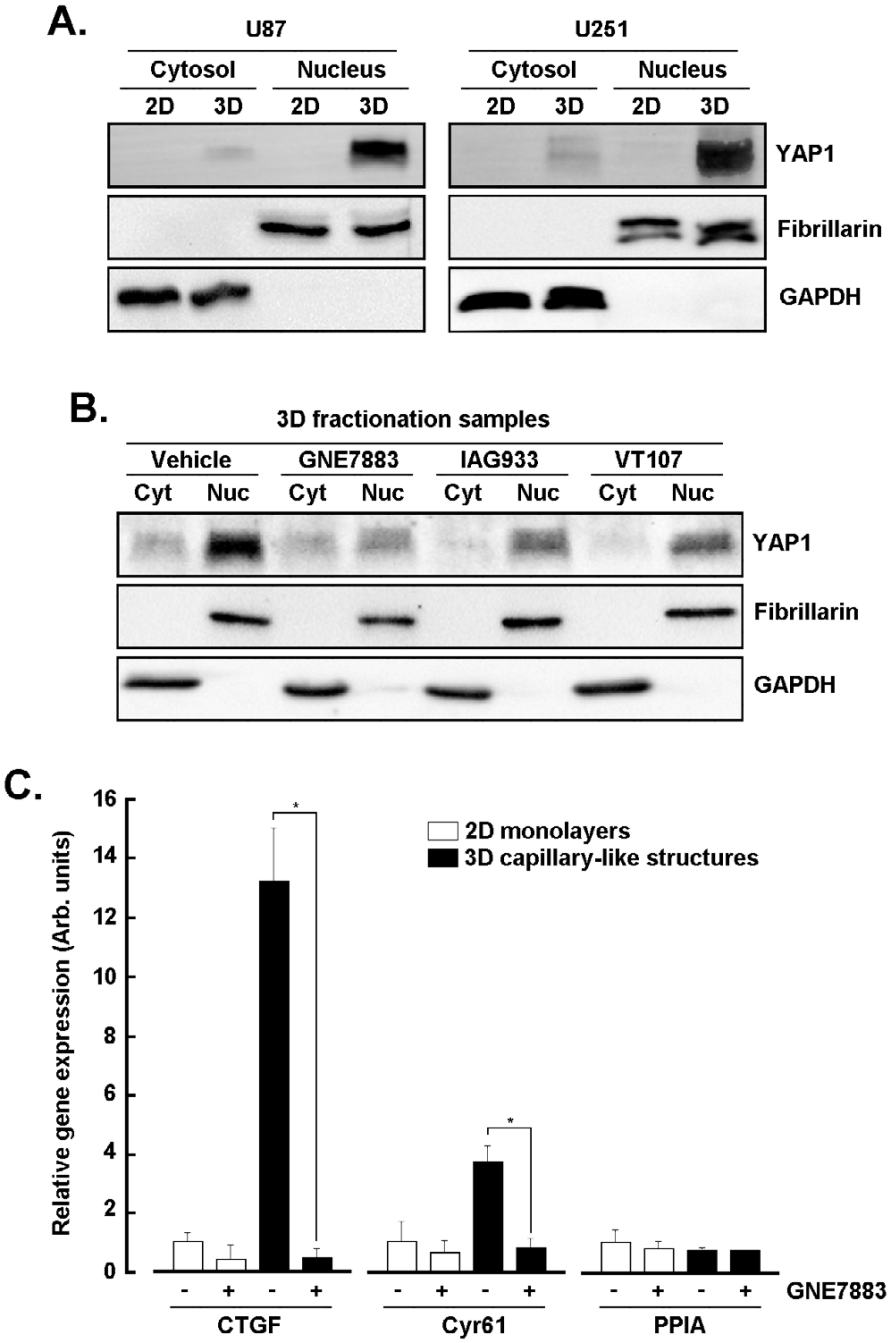
In vitro VM triggers nuclear YAP1 expression in U87 and U251 glioblastoma cells and is inhibited by Hippo pathway pharmacological targeting. (A) Human U87 and U251 glioblastoma cells were seeded either as monolayers (2D) or on top of Cultrex (3D) to generate capillary‐like structures. Subcellular fractionation was performed to isolate the cytosol and nuclear fractions from 2D or 3D cells. Representative blots for YAP1, Fibrillarin and GAPDH are presented from three independent fractionations. (B) Cytosolic (Cyt) and nuclear (Nuc) fractions were isolated from U87 glioblastoma cells seeded on Cultrex and treated with either vehicle or 10 μM of the indicated Hippo pathway inhibitors for 24 h. Representative blots for YAP1 and Fibrillarin are presented from two independent fractionations. (C) Total RNA was extracted from U87 cells cultured as in (A). RT‐qPCR was performed and relative gene expression of *CTGF*, *Cyr61* and *PPIA* normalised over *GAPDH*. Data presented are representative triplicates from two independent experiments (*p < 0.05).

Recent evidence established the utility of anti‐YAP/TAZ therapy in mouse models of metastatic melanoma whereby inhibition of VM appeared to prolong the survival of mice with melanoma brain metastases [39]. How in vitro VM‐mediated capillary‐like structure formation modulated YAP1 expression and transcriptional activity was next addressed. U87 and U251 GBM cells were therefore seeded either as monolayers (2D) or on top of Cultrex (3D) to generate capillary‐like structures. Subcellular fractionation was performed to isolate the cytosol and nuclear fractions from 2D or 3D cells. Nuclear YAP1 expression was found to significantly increase upon formation of VM in both of the cell lines tested (Figure 4A). When cytosolic and nuclear fractions were isolated from U87 GBM cells seeded on Cultrex and treated with either vehicle or the indicated Hippo pathway inhibitors, nuclear YAP1 expression was reduced (Figure 4B). Finally, transcript levels of two of the Hippo pathway downstream effectors *CTGF* and *Cyr61* previously reported to be induced upon in vitro VM [26] were assessed in U87 cells. *CTGF* and *Cyr61* were effectively found induced upon capillary‐like structure formation, and such induction was prevented by GNE7883 (Figure 4C). Collectively, this evidence suggests that nuclear YAP1 correlated with in vitro VM and contributed to the anti‐Hippo pathway pharmacological inhibition of VM through, in part, reduced transcriptional regulation of downstream effectors involved in 3D capillary‐like structure formation. The direct impact of YAP1 on VM was next addressed.

### 4.5 Silencing of YAP1 Alters In Vitro VM and Cell Migration in U87 and U251 Glioblastoma Cells

**FIGURE 5:**
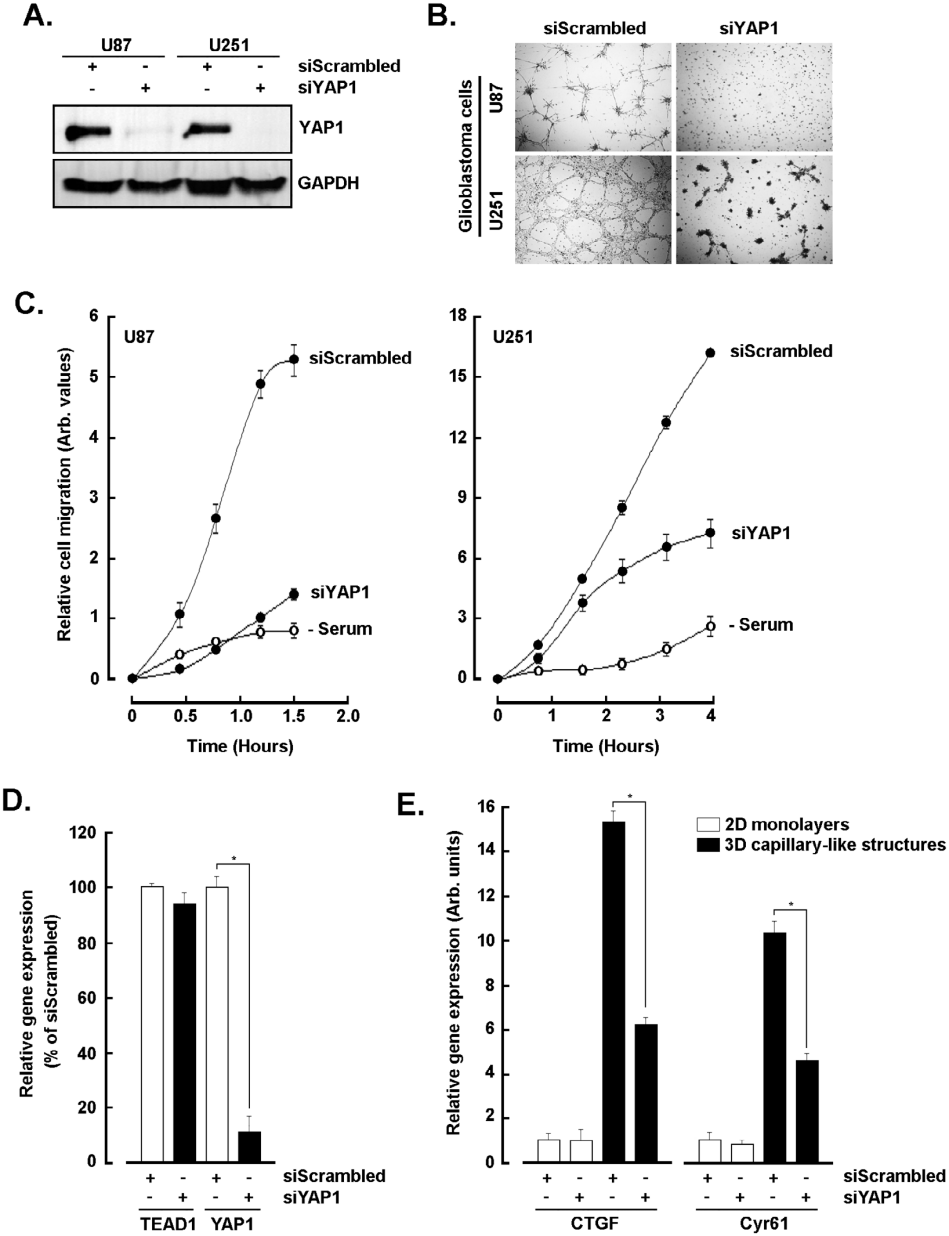
Silencing of YAP1 alters in vitro VM and cell migration in U87 and U251 glioblastoma cells. (A) Transient siRNA‐mediated gene silencing of YAP1 (siYAP1) or a non‐specific scrambled sequence (siScrambled) was performed in U87 and U251 glioblastoma cells. Total protein lysates were isolated, and representative immunoblots of YAP1 and GAPDH were presented out of three independent experiments. (B) Transfected U87 and U251 glioblastoma cells were seeded on top of Cultrex and 3D capillary‐like structures generated for 24 h. Representative phase contrast pictures were taken. (C) Transfected U87 and U251 glioblastoma cells were further assessed for real‐time cell migration using the xCELLigence system in response to serum (closed circles) in U87 (left panel) and U251 (right panel). Absence of serum was performed to assess cell migration under no chemotactic cues (open circles, − Serum). (D) Total RNA was extracted from U87 cell monolayers upon transient gene repression of *YAP1* and validated by RT‐qPCR. (E) Total RNA was extracted from U87 cells cultured as in B). RT‐qPCR was performed and relative gene expression of *CTGF* and *Cyr61* was assessed by RT‐qPCR. All RT‐qPCR data presented are representative triplicates from two independent experiments (*p < 0.05).

To assess the direct impact of YAP1 on VM, transient gene silencing was performed to specifically repress YAP1 and this was validated by immunoblotting at the protein level in both U87 and U251 cells (Figure 5A). Cells were next seeded on top of Cultrex and 3D structures found to be significantly altered as compared to control cells (siScrambled, Figure 5B). Transfected U87 and U251 cells were further assessed for real‐time chemotactic cell migration in response to serum. YAP1 repression was found to significantly inhibit chemotaxis in both cell models (Figure 5C). This observation provides evidence as to the direct impact of YAP1 on cell migration and ultimately supports its role on VM when Hippo pathway inhibitors prevent its nuclear expression. Finally, YAP1‐mediated transcriptional regulation of *CTGF* and *Cyr61* gene expression was explored by RT‐qPCR in U87 cells. Specific siRNA‐mediated repression of *YAP1* was confirmed (Figure 5D), and this abrogated the induction of *CTGF* and *Cyr61* upon capillary‐like structure formation (Figure 5E).

## 5 Discussion

YAP1 involvement in the progression of LGG to secondary GBM and its contribution to an aggressive brain tumour phenotype is increasingly established [40, 41]. As such, YAP1 overexpression was found to promote invasion and migration in GBM cells, correlating with poor patient prognosis [42]. A knockdown of YAP1 was further found to inhibit tumour growth [43, 44]. Beyond YAP's oncogenic transcriptional role, elevated levels of TAZ have been linked to the development of a Temozolomide‐resistance phenotype in human glioma cells [45]. Importantly, a growing body of literature also implicates YAP/TAZ activation in resistance to targeted therapies, chemotherapy, radiation and immunotherapies [46]. Implication of the Hippo/YAP1 signalling pathway in resistance to chemotherapy across various cancers was highlighted, including GBM and osteosarcoma where it increased resistance to drugs like methotrexate and doxorubicin [12, 47].

Given that *TAZ* levels did not significantly differ between healthy and tumour tissues, and that *TEAD1* could not discriminate between LGG and GBM tissues, we chose here to specifically assess the importance of the Hippo/YAP1 signalling in the highest aggressive type of brain cancer that GBM represents and where in silico analysis of YAP1 revealed significant and specific increases in clinically annotated GBM tissues. Key points highlighting such importance support the fact that Hippo pathway dysregulation leads to increased YAP/TAZ activity in GBM [2, 14, 48]. The crosstalk between the Hippo pathway and other signalling pathways, such as Wnt/β‐catenin and Notch, further amplifies GBM's resistance to therapy [12]. The Hippo pathway also influences the tumour microenvironment, including interactions with immune cells and their influence on VM [49, 50]. Accordingly, the presence of VM can affect the infiltration of immune cells into the tumour through the upregulation of immune checkpoints, such as CD28, CD86, BLTA, and CD40LG, which can inhibit the immune response [51]. Any strategies that can target the Hippo pathway involvement that leads to tumour immune escape may therefore circumvent chemoresistance and improve the overall effectiveness of treatment outcomes for GBM patients [35].

We further highlighted a novel role for the transcription factor YAP1 in the regulation of in vitro VM in GBM, a crucial process associated with chemoresistance. YAP1 regulates various genes involved in cell proliferation, survival and differentiation, including *CTGF*, which plays a role in cell adhesion, migration and proliferation, as well as *Cyr61*, which is associated with cell adhesion and angiogenesis. Additionally, YAP1 interacts with various transcription factors and signalling pathways, such as the TAZ and TEAD transcription factors family, to regulate these genes [52]. The YAP‐TEAD protein–protein interaction, which drives YAP oncogenic functions downstream of the Hippo pathway, now appears to include VM regulation. The consequences of a direct pharmacological disruption of the interface between YAP and TEADs by clinically relevant Hippo pathway inhibitors are here evidenced in vitro in several GBM cell models. Lastly, transient silencing of YAP1 (this study) or YAP‐inducible genes [26], as well as reduced GBM cell migration upon transient silencing of YAP, further confirms the validity of the Hippo pathway as a promising target for drug discovery. Our data further give support to those demonstrating that TAZ knockdown reduced SNB19 human glioma cell migration, likely due to impaired interaction with YAP1 [53].

Despite the potential of Hippo pathway inhibitors in cancer treatment, several limitations and challenges remain, particularly in treating GBM. As evidenced here from the cellular screen performed and differential response from four different established human GBM cell models, these will definitely include GBM tumour heterogeneity as this can affect how different tumours will respond to Hippo pathway inhibitors, making it challenging to develop a one‐size‐fits‐all treatment [2]. Recent advances in medicinal chemistry lead to potent Hippo pathway inhibitors and have demonstrated promising results in inhibiting YAP/TEAD transcriptional activity, altering in vitro VM, and reducing cancer cell migration [25, 26, 27, 28]. However, there still is also evidence that targeting the Hippo pathway could trigger adaptive resistance mechanisms [30]. Additionally, effective delivery of the Hippo pathway inhibitors to the tumour site, while minimising exposure to healthy tissues, also remains a significant challenge. This is particularly important for brain tumours like GBM, where the blood–brain barrier can impede drug delivery [54]. A better molecular understanding of the Hippo pathway in GBM will therefore lead to more effective future treatments and improve outcomes for patients suffering from this challenging to treat cancer.

## 6 Conclusions

Our study underscores the potential of targeting the Hippo pathway as a novel approach in cancer therapy, opening the door to further exploration of its role in VM‐mediated chemoresistance. Despite several drugs have been developed for the treatment of GBM, many of them have failed to secure approval or had shown limited efficacy during clinical trials largely due to their inability to target the chemoresistance phenotype of these rare brain cancers. Therefore, there is a high unmet need for new therapeutic strategies for GBM patients. Our findings reveal new pharmacological properties of clinically relevant small‐molecule YAP/TEAD inhibitors against VM processes. As VM can be triggered by numerous factors in GBM, such as hypoxia, inflammation, growth factors, and extracellular matrix components, it is also highly dependent on the tumour microenvironment and stage progression. Hence, treating VM‐related processes in GBM and other solid tumours with high VM activity presents a promising strategy to overcome such a considerable challenge.

## Author Contributions

**Marie‐Eve Roy:** conceptualization (supporting), data curation (lead), formal analysis (lead), investigation (lead), methodology (lead), supervision (lead), writing – original draft (lead), writing – review and editing (equal). **Rahil Elimam:** data curation (supporting), formal analysis (supporting), investigation (supporting), methodology (supporting), writing – original draft (supporting), writing – review and editing (supporting). **Alain Zgheib:** data curation (supporting), formal analysis (supporting), investigation (supporting), methodology (supporting), writing – review and editing (supporting). **Borhane Annabi:** conceptualization (lead), data curation (supporting), formal analysis (equal), funding acquisition (lead), project administration (lead), writing – original draft (supporting), writing – review and editing (lead).

## Conflicts of Interest

The authors declare no conflicts of interest.

## Data Availability

All data generated or analysed during this study are included in this published article and are available from the corresponding author upon reasonable request.
